# Thermal Imaging for Burn Wound Depth Assessment: A Mixed-Methods Implementation Study

**DOI:** 10.3390/jcm13072061

**Published:** 2024-04-02

**Authors:** Jesse de Haan, Matthea Stoop, Paul P. M. van Zuijlen, Anouk Pijpe

**Affiliations:** 1 Burn Center, Red Cross Hospital, Vondellaan 13, 1942 LE Beverwijk, The Netherlands; jdehaan@rkz.nl (J.d.H.); or m.stoop@amsterdamumc.nl (M.S.); or p.vanzuijlen@amsterdamumc.nl (P.P.M.v.Z.); 2 Plastic, Reconstructive and Hand Surgery, Amsterdam UMC Location Vrije Universiteit Amsterdam, De Boelelaan 1117, 1081 HV Amsterdam, The Netherlands; 3 Association of Dutch Burn Centers, 1941 AJ Beverwijk, The Netherlands; 4 Paediatric Surgical Center, Emma Children’s Hospital, Amsterdam UMC Location University of Amsterdam, Meibergdreef 9, 1105 AZ Amsterdam, The Netherlands; 5 Department of Plastic, Reconstructive and Hand Surgery, Red Cross Hospital, Vondellaan 13, 1942 LE Beverwijk, The Netherlands; 6 Amsterdam Movement Sciences, Tissue Function and Regeneration, 1081 HV Amsterdam, The Netherlands

**Keywords:** thermography, burn assessment, burn centre, healing potential, implementation

## Abstract

**Background:** Implementing innovations emerging from clinical research can be challenging. Thermal imagers provide an accessible diagnostic tool to increase the accuracy of burn wound depth assessment. This mixed-methods implementation study aimed to assess the barriers and facilitators, design implementation strategies, and guide the implementation process of thermal imaging in the outpatient clinic of a burn centre. **Methods:** This study was conducted between September 2022 and February 2023 in Beverwijk, The Netherlands. Semi-structured interviews with burn physicians guided by the Consolidated Framework for Implementation Research (CFIR) were conducted to identify barriers and facilitators. Based on the barriers, implementation strategies were developed with the CFIR-ERIC Matching Tool, and disseminated to support the uptake of the thermal imager. Subsequently, thermal imaging was implemented in daily practice, and an iterative RE-AIM approach was used to evaluate the implementation process. **Results:** Common facilitators for the implementation of the thermal imager were the low complexity, the relative advantage above other diagnostic tools, and benefits for patients. Common barriers were physicians’ attitude towards and perceived value of the intervention, the low compatibility with the current workflow, and a lack of knowledge about existing evidence. Six implementation strategies were developed: creating a formal implementation blueprint, promoting adaptability, developing educational materials, facilitation, conducting ongoing training, and identifying early adopters. These strategies resulted in the effective implementation of the thermal imager, reflected by a >70% *reach* among eligible patients, and >80% *effectiveness* and *adoption*. Throughout the implementation process, compatibility, and available resources remained barriers, resulting in low ratings on RE-AIM dimensions. **Conclusions:** This study developed implementation strategies based on the identified CFIR constructs that impacted the implementation of a thermal imager for burn wound assessment in our outpatient clinic. The experiences and findings of this study could be leveraged to guide the implementation of thermal imaging and other innovations in burn care.

## 1. Introduction

Implementing evidence-based interventions in burn care and other fields is challenging, even in the face of compelling evidence [1]. It is imperative to identify and address these challenges in order to implement new interventions and produce health benefits for patients. The field of implementation science seeks to identify and address the barriers that slow or halt the uptake of proven health interventions and evidence-based practices [2].

Correctly assessing burn wounds is vital in determining their depth and potential for healing, which are both significant factors to consider when choosing a treatment approach [3]. Burn wounds that are expected to epithelialise in less than 21 days can often be conservatively managed with topical creams, whereas burn wounds that will epithelialise in more than 21 days may cause hypertrophic scarring and require surgical treatment [4]. Therefore, misclassifying burn wound healing potential can result in undertreatment or unnecessary surgery. Thus far, clinical assessment is the most frequently used method for assessing burn depth and healing potential, with a reported accuracy of around 50–71.4% between days two and five postburn [5,6,7].

Several objective diagnostic tools have been studied to improve the accuracy of clinical burn wound depth assessment. To date, laser Doppler imaging (LDI) is an accurate and commonly used tool in specialised burn centres, with studies reporting over 95% accuracy if performed between days two and five postburn [8]. Despite its high accuracy, LDI has several drawbacks. The device is expensive, large, inconvenient to relocate, and time-consuming to use. Additionally, patients need to lie still during measurements, as movements may generate measurement artefacts. This can particularly be challenging during the burn wound assessment of children [9]. 

As opposed to LDI, thermal imagers are relatively cheap, easy to use, small, connectable to mobile phones and tablets, and able to capture images in seconds [10]. These devices may be particularly valuable in settings with a short consultation time, or when LDI is unavailable or impractical to use. Several thermal imagers have been validated for burn wound assessment and proven to be reliable [11]. Thermal imagers utilise infrared emission to measure the skin’s surface temperature. By measuring the temperature difference between a burn wound and healthy skin (ΔT), a prediction can be made of the burn wound’s healing potential [10,12,13,14,15].

Given the results on the validity and reliability of thermal imaging for burn wound depth assessment, a consecutive step would be its implementation into clinical practice. Therefore, we conducted a mixed methods implementation study on thermal imaging for burn wound assessment in the outpatient clinic of our burn centre. The aim of our study was to: (1) identify barriers and facilitators to the implementation of thermal imaging; (2) develop and disseminate implementation strategies to promote adoption; and (3) evaluate implementation outcomes throughout the implementation process.

## 2. Materials and Methods

### 2.1. Study Design

**Figure 1:**
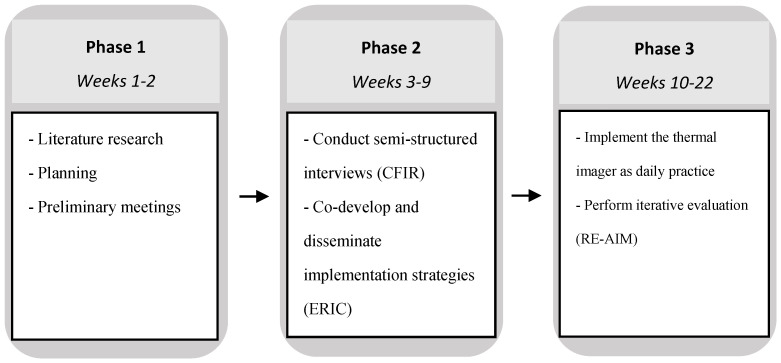
Study design.

This mixed-methods implementation study took place over a 22-week period, and consisted of three phases: (1) planning phase, including a literature search; (2) the assessment of barriers and facilitators, followed by the design and testing of implementation strategies; (3) begin implementation as normal daily practice with iterative evaluation of implementation outcomes (Figure 1). The study was described according to the Standards for Reporting Implementation Studies (STaRI) checklist [16].

### 2.2. Study Population and Setting

This study was conducted between September 2022 and February 2023 in the burn centre of the Red Cross Hospital, Beverwijk, The Netherlands. The study was conducted according to the guidelines of the Declaration of Helsinki, and approved by the director of the Burn Center, Red Cross Hospital, Beverwijk, The Netherlands. The burn centre treats both children and adults, and consists of an intensive care unit (ICU), a burn ward, and an outpatient clinic. Currently, the LDI is used for burn wound assessment in our burn ICU and ward. The scans are made by nurses. However, burn wound assessment in the outpatient clinic currently relies on clinical evaluation due to the time-consuming nature of the LDI and infrastructural limitations. There was a thermal imaging device in our centre but this was not widely used, and hence, the project was initiated to consider the usage in more detail. In total, the burn centre physician team consisted of four burn physicians, two surgeons, and one burn physician in training. These were the intended users of the thermal imager, and the participants in this study. Data on patients visiting the outpatient clinic during the study period were collected anonymously: throughout the implementation process, quantitative outcome data were extracted from the electronic health record and documented on a group level. See the Supplementary File for the patient eligibility criteria.

### 2.3. Thermal Imaging Camera and Imaging Procedure [10,12,15]

The thermal imager used in this study was the FLIR ONE Pro thermal imager (FLIR Systems, Inc., Wilsonville, OR, USA) attached to an iPad mini (Apple, Inc. Cupertino, CA, USA) with a lightning connector. The dimensions of the device are 67 × 14 × 34 mm (height × width × depth), and it weighs 36.5 g. The device consists of a thermal camera with a resolution of 160 × 120 pixels, and a VGA camera with a resolution of 1400 × 1080 pixels. The images produced by these cameras are combined, resulting in a thermal image that is able to detect temperature differences as small as 0.05 °C [17]. The device needs a minimum of 15 centimetres to focus. There is no maximum distance for capturing thermal images; however, the resolution of the thermal image will reduce with larger distances. See the Supplementary File for the imaging procedure.

### 2.4. Study Design

#### 2.4.1. Phase 1: Planning

First, a thorough investigation of the different theories, models, and frameworks (TMF’s) that are available in dissemination and implementation research was conducted. A combination of the CFIR and the iterative RE-AIM approach was chosen to capitalise on the complementary strengths of each framework [18,19]. While CFIR seeks to identify contextual factors impacting implementation processes, the iterative RE-AIM approach facilitates a rapid and comprehensive evaluation of the implementation outcomes. This dual approach allows for a more nuanced and thorough examination of our intervention’s effectiveness within the study’s constrained timeframe. Second, a literature search was performed in September 2022 to gather the latest scientific information about thermography for assessing burn wound healing potential. All articles that investigated thermographic imaging for the assessment of burn wound healing potential were included. One additional study was identified besides those included in the recently published systematic review by Dang et al. [11,13]. Third, ΔT cut-off values were extracted from the study by Carrière et al. [15]. This was the only study that developed ΔT cut-off values based on a 95% specificity, resulting in a high number of true positives. A higher specificity value would result in a lower sensitivity value, and thus reduce the thermal imager’s clinical usefulness. Fourth, two preliminary meetings were held with the physicians who were going to use the thermal imager in daily practice. During these meetings, the implementation team gave a brief introduction to implementation science, explained the rationale for using the thermal imager in the outpatient clinic, and revealed the implementation plan.

#### 2.4.2. Phase 2: Barriers and Facilitators, Implementation Strategies

In this phase, implementation strategies were developed based on the barriers identified during semi-structured interviews with the burn physicians and surgeons working at the outpatient clinic, as they were going to be the primary users of the thermal imager. A semi-structured interview guide was developed with questions adapted from the CFIR Interview Guide [20]. The focus of these interviews was to identify barriers and facilitators and estimate their impact on the implementation process. The interviews were conducted individually to prevent the influence from of group dynamics. All interviews were audio recorded, transcribed verbatim, and analysed with MAXQDA 2022 (VERBI Software, Berlin, Germany). Responses to the interview questions were coded deductively, using the CFIR constructs with their corresponding definitions as coding framework [21]. CFIR constructs are different aspects or factors that can influence the success of implementation.

Once all data were organised into CFIR constructs, two researchers rated the constructs by applying specific rating criteria [22]. Potential barriers identified during the interviews were matched to implementation strategies with the CFIR-ERIC Implementation Strategy Matching Tool [23]. With this tool, negatively rated CFIR constructs can be entered, resulting in a list of implementation strategies derived from the Expert Recommendations for Implementing Change (ERIC) list of strategies [24]. The strategies that were considered applicable for this specific healthcare context were selected. These strategies were further specified using Proctor’s recommendations for specifying and reporting implementation strategies [25].

Prior to the start of Phase 3, selected implementation strategies were disseminated through email and elucidated verbally. Subsequently, the implementation strategies were applied over the course of the implementation process, where timing was dependent on the strategy.

#### 2.4.3. Phase 3: Implementation

In Phase 3, the thermal imager was implemented in the outpatient clinic as ‘’normal’’ daily practice (see Supplementary File for eligibility criteria and imaging procedure). During this phase, iterative RE-AIM was used as guidance to enable rapid-cycle evaluation [26]. For definitions of the five RE-AIM dimensions, see Appendix A. Throughout the implementation process, quantitative outcome data on RE-AIM dimensions was documented on a weekly basis. Data on the dimensions Reach, Effectiveness, and Adoption were extracted from the electronic health record (EHR). Documenting these outcome measures weekly allowed the implementation team to assess progress on RE-AIM dimensions over time. This progress was evaluated during two evaluation cycles. The first evaluation cycle was conducted after 7 weeks, the second evaluation cycle after 13 weeks, which was the end of the study period.

The evaluation cycles consisted of the two following steps: (1) All participants were informed about the iterative RE-AIM evaluation process by email. Additionally, they received a link to an online rating sheet, and were asked to individually rate each RE-AIM dimension in terms of importance and progress to date using a five-point Likert scale. Researchers had the ability to identify which participant completed each rating sheet. Participants were asked to rate progress based on the objective quantitative outcome data, as well as subjective experiences. Ratings could be substantiated with comments and examples. (2) The scores of the rating sheets were summarised in charts and distributed among the participants. Each participant received a copy of their own ratings and the team summary. They were asked to individually review the results, and particularly focus on imbalances between importance and progress to date. Based on these summaries and personal experiences, a team discussion was held with the aim of identifying RE-AIM dimensions that needed addressing. Due to part-time schedules, there was no date available that allowed all participants to attend a single meeting. Therefore, two separate meetings were held. After the relevant RE-AIM dimensions were selected, team members were requested to formulate goals and strategies to improve implementation outcomes of these specific dimensions. Goals were formulated according to the SMART principle (Specific, Measurable, Achievable, Relevant, Time-bound). During the second evaluation cycle, the team discussion was predetermined to focus on the dimension maintenance. In addition, participants were asked what they needed for long-term sustainment instead of formulating SMART goals and strategies.

The team discussions were recorded and transcribed verbatim. Subsequently, MAXQDA 2022 was used for thematic analysis. Transcripts were reviewed multiple times in order to identify themes, inductively code the data, and develop a codebook. A separate codebook was developed for each evaluation cycle.

## 3. Results

### 3.1. Barriers and Facilitators

Six out of a total of seven participants were interviewed during September and October 2022. All interviews were in-person and ranged from 25 to 35 min. One physician could not be interviewed due to an international internship. An overview of the assigned ratings of CFIR constructs is provided in Table 1. Opinions and arguments from interviewees are provided in Table 2.

### 3.2. Implementation Strategies

The CFIR-ERIC matching tool provided a list of implementation strategies to address the barriers that were identified during the interviews. Using this tool, the six following implementation strategies that fitted this specific healthcare context were selected (Table 3): creating a formal implementation blueprint, promoting adaptability, developing educational materials, facilitation, conducting ongoing training, and identifying early adopters.

### 3.3. Evaluation of Implementation

Table 4 provides an overview of the quantitative data that were collected during the implementation process. In total, the participants completed five rating sheets (Table 5).

#### 3.3.1. First Evaluation Cycle

During the first seven weeks, the first evaluation cycle, 49 patients presented at the outpatient clinic, of whom 17 patients presented between day two and five postburn (35%). Of these 17 patients, 13 patients (76%) with a total of 16 burn wounds were imaged. During the first five weeks, four children (24%) were excluded from participation due to a lack of consensus among physicians regarding the use of thermal imaging for paediatric burn patients. Of the 16 burn wounds that were imaged, twelve (80%) were classified correctly, and three burn wounds (20%) could not be classified (−0.6 °C ≤ ΔT ≤ 0.4 °C). Four out of seven participants used the thermal imager at least once. The three participants who did not take any images during the first seven weeks worked at the outpatient less frequently. When they did work at the outpatient clinic, no patients attended between days two and five postburn. None of the participants refused to use the thermal imager.

Between 29 December 2022 and 3 January 2023, the implementation team facilitated two team discussions with six participants, lasting 30 min each. One participant was unable to attend the meetings. From these discussions, two key themes emerged: less uncertainty for patients and lack of experience. Participants thought that the thermal imager helped them in making firmer statements about the expected healing potential, resulting in less uncertainty for patients. Additionally, it supported clinical decision making and helped them in visualising their explanation to the patient. Some participants, however, shared that the results of the thermal imager were often identical to their clinical assessment. Although effectiveness was perceived as good, they mentioned that the added value of the device could therefore be limited. Based on the provided quantitative data and subjective experiences, all participants concluded that the reach of the intervention was low. They stated that this was mainly caused by the short window of opportunity from days two to five postburn, as most patients presented outside this time window. As a consequence, adoption rates were low and learning curves too flat. Since reach was deemed to be difficult to improve, the team goals and action plans focused on adoption. Both teams proposed to take a thermal image simultaneously with the LDI scan of all patients that are admitted to the burn ward, and between days two and five postburn. This would expand use amongst the participants, and allow them to become acquainted with the device. Furthermore, participants proposed to put a reminder in the patient’s EHR after imaging them in order to evaluate the results during the patient’s follow-up appointment, to become conversant with the interpretation of the thermal images.

#### 3.3.2. Second Evaluation Cycle

After 13 weeks, *reach*, *effectiveness*, and *adoption* were 71%, 82%, and 86%, respectively. Between weeks 7 and 13, six more eligible patients were not imaged, resulting in a total of 10 patients (29%). Three patients were not imaged as the thermal imager was in use by another participant on that specific moment, and participants forgot to use the thermal imager in two other cases. One patient had an impaired vascular status and was excluded from imaging. The two falsely classified burn wounds were initially categorised as requiring more than 14 days to epithelialise but epithelialised within 14 days. Two healing potentials remained unknown as these patients did not attend their follow-up meeting. One participant had not been able to use the thermal imager in practice since they were scheduled to work just once and then no eligible patients presented.

Between 7 February 2023 and 14 February 2023, the implementation team facilitated a team discussion focussed on the dimension *maintenance* with four participants. Three participants were unable to attend this meeting due to a high clinical workload. The following key themes emerged: workload and optimised software. Regarding workload, the participants experienced the steps and time needed to upload a thermographic image into the patient’s EHR as excessively long, making it a challenge to accomplish within the 10 min timeframe allocated per patient consultation. Additionally, participants felt that the software application was not optimal for use in a healthcare context. They preferred an immediate result, rather than having to select the appropriate regions of interest (ROI) and reference area in the burn wound, and manually calculate the difference between those. Additionally, participants experienced the limitations of analysing previously made thermographic images uploaded in the EHR, since a previously assigned ROI could not be adjusted or modified. In these cases, participants could only view the ∆T and healing potential for the specific areas that had been previously assigned.

#### 3.3.3. Long-Term Needs 

After the second, final evaluation cycle, participants described four key needs for long-term sustainment of using the thermal imager in the outpatient clinic. First, they suggested that the thermal imager should be validated for a wider time range to increase the *reach*. Second, they expressed a desire for software that could provide an immediate result, eliminating the need for the manual calculation of the ∆T for every region of interest. Third, they wanted the time investment to be lower, particularly for uploading the thermographic images in the EHR. Fourth, participants noted that it was important that they would take ownership of the intervention since the active support phase facilitated by the implementation team was over.

After the formal study period had ended, we learned that the *reach* of the thermal imager dropped and that the device was not used as much as during the study period when facilitation was present.

## 4. Discussion

This mixed-methods study used a combination of implementation science frameworks to perform multi-contextual analysis, develop implementation strategies, assess implementation outcomes, and guide the implementation process of a thermal imager in the outpatient clinic of a specialised burn centre. Pre-implementation, 10 barriers and 8 facilitators were identified that could impact the implementation process. To overcome these barriers, six implementation strategies were developed and disseminated. These strategies resulted in the effective implementation of the thermal imager, reflected by >70% *reach* among eligible patients, and >80% *effectiveness* and *adoption*. However, some barriers persisted throughout the implementation process, affecting long-term sustainment.

Several facilitators identified in this study were located in the CFIR domain *intervention characteristics*. Participants considered the thermal imager to have a *relative advantage* over other diagnostic tools for burn wound assessment in the outpatient clinic, due to its low *complexity* and fast speed of use. However, the factor considered to be the most important was the extent to which the thermal imager could benefit patients. Some participants observed that patients perceived the visual results of LDI as comforting, and believed that the thermal imager could elicit a similar response. To our knowledge, no studies have investigated how patients experience viewing the diagnostic images of their burn wounds. However, research in other fields suggests that it might give patients a better understanding of their diagnosis, and increase their trust in the healthcare provider [27]. Therefore, obtaining feedback from patients might have been a helpful implementation strategy to increase provider buy-in for the thermal imager.

The barriers to implementing the thermal imager were mainly situated in the *inner setting* domain. Notably, the *Tension for Change* was low, which did not align with our expectations. LDI has become the standard of care in our burn ward, and in the past, physicians frequently mentioned that every patient in the burn ward should be imaged. However, some believed that few mistakes were being made during clinical assessment, and mentioned that they would ultimately rely on their clinical assessment. A study that used a survey to investigate barriers to the use of LDI reported similar findings [1]. In this study, only 12.5% of the responders relied on LDI results in case of a discrepancy between LDI and clinical assessment. Discussing new images during educational meetings could have been a valuable implementation strategy to reduce this barrier *knowledge and beliefs*, as this would provide an opportunity to showcase instances where the thermal imager proved to be helpful.

Although efforts were made to reduce the time needed to analyse a thermographic image, and upload this in the patient’s EHR, the construct *available resources* remained a barrier. This was partially represented by the low ratings on the dimension *implementation*. However, increasing staffing or time per consultation were not considered viable implementation strategies. Additionally, other healthcare settings with a short consultation time will likely face similar challenges. Southerland et al. experienced the same issue, and suggested further reducing the time and effort required for utilising the intervention, as this may be a more practical solution than hiring additional staff members [28]. *Compatibility* remained a barrier throughout the implementation process, which likely resulted in the low ratings on *reach* and *adoption*. Several participants noted that the *reach* of the thermal imager was low due to its time window of days two to five postburn. However, it is worth noting that, in a healthcare setting in which the majority of patients present between days two to five postburn, *reach* is presumably higher. There are several studies conducted that investigated the validity of thermal imagers outside of this time window. A validation trial of the FLIR T300 thermographic imager found no correlation between absolute temperatures and burn depth on days one and two postburn [29]. Ganon et al. investigated the validity of the FLIR ONE by comparing the absolute temperatures to wound healing time on three different time points (T1 = day 1–3, T2 = day 4–7, T3 = day 8–10) [14]. Although the area under the curve (AUC) on these time points was good (0.787, 0.700, 0.968, respectively), the reported ∆T cut-off values were based on a 100% specificity. Consequently, outliers may have had a significant influence on the cut-off values, reducing the sensitivity value, and thus the thermal imager’s usefulness in clinical practice. It is worth noting that there was a discrepancy between the ratings participants gave on the dimension *effectiveness* and the quantitative data that were collected. The dimension *effectiveness* received weak ratings, even though almost all burn wounds were classified correctly. Therefore, it seems that these low ratings were a consequence of a limited perceived added value of the thermal imager above clinical evaluation alone. Not unexpectedly, ratings on *reach*, *adoption*, and *implementation* remained low. This was likely because we were unable to further reduce the time investment to upload the image in the EHR, and widen the time window in which the thermal imager could be used.

Although the findings of this study are specific to the healthcare setting in which the study was conducted, they have important implications. The identified barriers contribute to a clearer understanding of what adaptations the thermal imager might need, and therefore provide recommendations for future research that aims to facilitate its uptake in clinical practice. Special attention should be given to address the barrier *compatibility*, to ensure the high *reach*. By doing so, participants are more likely to become acquainted with the device, and adopt the device in their work process. Second, these results build on the existing opinion that the thermal imager has a *relative advantage* in terms of accessibility and *complexity* [10,13,14,15]. However, a software application that provides an LDI-like colour scheme based on the reference area that is selected by the physician could significantly improve the usability and apprehensibility of the thermal images. This could address the barrier *available resources*, and further reduce the *complexity* of the device. Third, the implementation strategies from this study can be utilised to facilitate the implementation of thermographic imaging in other healthcare settings that seek to implement a fast diagnostic tool for burn wound assessment.

Our study has several strengths, including the use of internationally accepted implementation methodologies, the development of implementation strategies based on pre-implementation identification of barriers, and the iterative evaluation of outcomes throughout the implementation process. However, limitations should be considered. First, the study period of this implementation period was relatively short compared to other implementation studies [30,31], which may have resulted in lower-rated implementation outcomes. Second, the iterative evaluation was conducted in two separate groups, as there was no date available that could include all participants. This may have hindered participants from learning from others’ positive and negative experiences.

## 5. Conclusions

In conclusion, this study implemented a thermal imager in a real-world specialised burn centre, and utilised mixed methods for a comprehensive understanding of the implementation process. However, several challenges remain, affecting long-term sustainment. The findings of this study could be leveraged to guide future research, and promote the adaptability of thermal imaging in burn wound assessment. Furthermore, developed implementation strategies could be tailored and utilised to facilitate implementation in other organisations.

## Figures and Tables

**Table 1:** CFIR construct rating matrix.

CFIR Construct	Rating
**I. Intervention Characteristics**	
A. Innovation source	0
B. Evidence strength and quality	−2
C. Relative advantage	+2 *
D. Adaptability	0
E. Trialability	M
F. Complexity	+2 *
G. Design quality and packaging	M
H. Cost	0
**II. Outer Setting**	
A. Patient needs and resources	+1 *
B. Cosmopolitanism	0
C. Peer pressure	X
D. External policies and incentives	M
**III. Inner setting**	
A. Structural characteristics	0
B. Networks and communications	−1 *
C. Culture	+2
D. Implementation climate	+1
1. Tension for change	−1 *
2. Compatibility	−2 *
3. Relative priority	−1 *
4. Organisational incentives and rewards	M
5. Goals and feedback	0
6. Learning climate	M
E. Readiness for implementation	−1
1. Leadership engagement	M
2. Available resources	−1
3. Access to knowledge and information	−2
**IV. Characteristics of individuals**	
A. Knowledge and beliefs about the intervention	−1 *
B. Self-efficacy	+2 *
C. Individual stage of change	+2
D. Individual identification with organisation	M
E. Other personal attributes	+2
**V. Process**	
A. Planning	−2
B. Engaging	0
1. Opinion leaders	M
2. Formally appointed internal implementation leaders	0
3. Champions	M
4. External change agents	M
5. Key stakeholders	0
6. Innovation participants	0
C. Executing	M
D. Reflecting and evaluating	M

−2 = strong barrier; −1 = weak barrier; 0 = neutral; +1 = weak facilitator; +2 = strong facilitator; M = missing; * = mixed comments; X = the interviewee made comments that were purely descriptive. There were no statements that described influence of the construct on implementation.

**Table 2:** Opinions and arguments from interviewees.

**Intervention Characteristics**		
**Construct**	**Barrier/Facilitator**	**Argumentation**
*Evidence strength and quality*	Strong barrier	- Two participants mentioned that they contributed to previous publications about the thermal imager. However, they were unfamiliar with the current state of the evidence, and requested more information about the reliability and validity of the thermal imager.
*Relative advantage*	Strong facilitator	- Most participants considered the thermal imager as more easily accessible and faster than the LDI, and therefore more suitable for the outpatient clinic. - One participant preferred the implementation of the LDI above the thermal imager, and suggested a comparative implementation trial.
*Complexity*	Strong facilitator	- All participants were in agreement that the device was uncomplicated and easy to use.
**Outer Setting**		
**Construct**	**Barrier/Facilitator**	**Argumentation**
*Needs and resources of those served by the organisation*	Weak facilitator	- Thermographic imaging might give patients more certainty about the prognosis of their burn wound. - Early and accurate assessment of healing potential could enable earlier surgical intervention. - Inaccurate results could result in a false sense of security. - Taking and analysing thermographic images might result in longer, and thus more uncomfortable dressing changes.
**Inner Setting**		
**Construct**	**Barrier/Facilitator**	**Argumentation**
*Networks and communications*	Weak barrier	- Participants mentioned it was difficult to organise all participants together due to part-time schedules.
*Culture*	Strong facilitator	- Participants experienced the culture in the burn centre as research-minded. - The majority of the burn centres’ employees cooperate in research and embrace innovation.
*Implementation climate*	Weak facilitator	- All participants perceived their colleagues’ receptivity to the intervention as positive, and thought that they were going to use the thermal imager in daily practice.
*Tension for change*	Weak barrier	- Participants shared that it was important to combine clinical assessment with a diagnostic tool to increase the overall accuracy of burn wound assessment. - Most participants felt that the implementation of a diagnostic tool in the outpatient clinic was not imperative. Participants mentioned that these burn wounds were smaller, and that the relative gain of early surgery would therefore be less than for patients admitted to the burn ward. - Some participants believed that few errors were being made during the clinical evaluation of burn wounds, and that the added value of thermal imaging was therefore limited.
*Compatibility*	Strong barrier	- Participants perceived the window of opportunity between days two and five postburn as short. They argued that patients frequently visit the outpatient clinic outside this time frame, suggesting that the thermal imager might not be applicable for all patients. - Participants wondered whether the thermal imager fitted in their current workflow. Lack of time was perceived as a prominent barrier to the implementation of the thermal imager.
*Relative priority*	Weak barrier	- One participant shared that physicians might experience change fatigue due to the amount of innovations that are implemented on a daily basis. - All participants indicated that there were no ongoing activities, studies, or initiatives that could interfere with the implementation process.
*Readiness for implementation*	Weak barrier	- Participants experienced a high workload.
*Available resources*	Weak barrier	- Participants mentioned lack of time as a barrier to taking thermal images.
*Access to knowledge and information*	Strong barrier	- All participants requested more practical information, such as a protocol, a description on how to use the device, and training.
**Characteristics of Individuals**		
**Construct**	**Barrier/Facilitator**	**Argumentation**
*Knowledge and beliefs about the innovation*	Weak barrier	- Participants shared that they primarily considered the thermal imager as an add-on test to clinical evaluation, and would ultimately trust their clinical assessment.
*Self-efficacy*	Strong facilitator	- The majority of participants were confident in their abilities to take and analyse a thermographic image.
*Individual stage of change*	Strong facilitator	- All participants were willing to use the thermal imager during the study period.
*Other personal attributes*	Strong facilitator	- Participants emphasised the high priority they placed on research, expressing motivation, and a commitment to innovative work.
**Process**		
**Construct**	**Barrier/Facilitator**	**Argumentation**
*Planning*	Strong barrier	- The planning of the implementation process remained unclear to the majority of participants.

**Table 3:** Expert recommendations for implementing change (ERIC) implementation strategies.

ERIC Strategy	To Address Barrier	Description	Justification
**Create a formal implementation blueprint**	- Planning	During the pre-implementation phase, the implementation team created a formal implementation blueprint. This blueprint contained a timeframe, milestones, and important dates (e.g., evaluation cycles), and was disseminated via email.	Participants requested additional practical information, such as a planning.
**Promote adaptability**	- Compatibility	The implementation team conducted two meetings with the hospitals’ ICT specialists during the pre-implementation phase. During these meetings, they identified the usage of a shared disk as the least time-consuming way in which the thermal images can be transferred to the electronic health record (EHR), and linked to the correct patient.	Various participants indicated that the easy transferability of thermal images to the EHR is essential for successful implementation. In addition, lack of time was a prominent barrier to the implementation process.
**Develop educational materials**	- Evidence strength and quality - Access to knowledge and information - Knowledge and beliefs about the intervention	During the pre-implementation phase, the implementation team developed and disseminated educational materials. Educational materials included the following: (1) guidelines on how to use the thermal imager, (2) an overview of evidence about the thermal imager, and (3) a reference card containing inclusion criteria, exclusion criteria, ΔT cut-off values, and a step-by-step guide on how to use the device.	All participants requested practical information (e.g., how to start the device, how to obtain results, who are going to use the device, how to get results in the EHR).
**Facilitation**	- Networks and communications - Compatibility - Knowledge and beliefs about the intervention - Planning	During the implementation phase, implementation team members were present in the outpatient clinic to support the implementers. This allowed for interactive problem solving.	The quick and effective countering of emerging problems is essential, as these problems may impede the implementation process. In addition, facilitation can promote relationships between the implementation team and participants.
**Conduct ongoing training**	- Access to knowledge and information - Planning	The implementation team conducted ongoing training during the implementation phase.	Conducting ongoing training enhances the implementation fidelity and speed of usage.
**Identify early adopters**	- Evidence strength and quality - Knowledge and beliefs about the intervention	During the implementation phase, the implementation team identified the early adopters of the thermal imager to learn from their experiences.	Identifying early adopters can provide the implementation team with valuable information on why some participants are more successful in implementing the intervention than others. This information can be used to foster implementation amongst late adopters.

All strategies were specified according to Proctor’s recommendations for specifying and reporting implementation strategies [25].

**Table 4:** Quantitative data of RE-AIM outcome measures.

	At 7 Weeks (Cycle 1)	At 13 Weeks (Cycle 2)
**Reach**		
Number of patients that presented at the outpatient clinic (*n*) ^a^	49	102
Number of patients presenting between days 2 and 5 postburn (*n*)	17	35
Number of patients imaged (*n*)	13	25
Proportion of patients that were imaged compared to the number of patients that presented at the outpatient clinic (*%*)	26.5%	24.5%
Proportion of patients that were imaged compared to the number of patient presenting between days 2 and 5 postburn (*%*)	76.5%	71.4%
Number of imaged burn wounds (*n*)	16	30
**Effectiveness** ^b,c^		
Number of correctly classified burn wounds (*n*)	12	23
Number of falsely classified burn wounds (*n*)	0	2
Number of inconclusive results (*n*) ^c^	3	3
Healing potential unknown (*n*)	1	2
**Adoption**		
Number of staff that used the thermal imager (*n*)	4/7	6/7

^a^ First presentation. ^b^ Based on the information from the electronic health record (EHR). ^c^ ΔT < −2.3 °C = healing potential (HP) ≥ 21 days. ΔT < −0.6 °C = HP ≥ 14 days. −0.6 °C ≤ ΔT ≤ 0.4 °C = inconclusive result. ΔT > 0.4 °C = HP < 21 days. ΔT > 0.6 °C = HP < 14 days [10,12,15].

**Table 5:** Rating sheet results.

RE-AIM Dimension	Rating	At 7 Weeks (Cycle 1)	At 13 Weeks (Cycle 2)
**Reach**	Rating on importance	4 (3.5–5)	4 (3–4.5)
	Rating on satisfaction with progress	2 (1.5–3)	2 (2–2.5)
**Effectiveness**	Rating on importance	5 (4.5–5)	5 (4–5)
	Rating on satisfaction with progress	3.5 (2.5–4)	2 (2–3.5)
**Adoption**	Rating on importance	4 (3–5)	4 (3.5–4.5)
	Rating on satisfaction with progress	2 (1.5–3.5)	2 (1.5–2.5)
**Implementation**	Rating on importance	5 (3–5)	4 (3–5)
	Rating on satisfaction with progress	2 (2–3)	2 (2–2.5)

All ratings are reported as median (i.q.r.).

## Data Availability

The data presented in this study are available upon reasonable request from the corresponding author.

## Supplementary Materials

The following supporting information can be downloaded at: https://www.mdpi.com/article/10.3390/jcm13072061/s1. Table S1. ΔT cut-off values and associated healing potential (HP) categories. Table S2. Criteria used to assign ratings to CFIR constructs. Figure S1. This thermographic image had a ΔT of more than 0.6 °C, corresponding with an expected healing potential of <14 days.

## Appendix A. Definitions RE-AIM Dimensions [19]

**Reach:** The absolute number, proportion, and representativeness of individuals who are willing to participate in a given initiative, intervention, or program. **Effectiveness:** The impact of an intervention on important outcomes, including potential negative effects, quality of life, and economic outcomes. **Adoption:** The absolute number, proportion, and representativeness of settings and intervention agents (people who deliver the program) who are willing to initiate a program. **Implementation:** At the setting level, implementation refers to the intervention agents’ fidelity to the various elements of an intervention’s protocol, including the consistency of delivery as intended and the time and cost of the intervention. At the individual level, the implementation refers to clients’ use of the intervention strategies. **Maintenance:** The extent to which a program or policy becomes institutionalised or part of the routine organisational practices and policies. Within the RE-AIM framework, maintenance also applies at the individual level. At the individual level, maintenance has been defined as the long-term effects of a program on outcomes after 6 or more months after the most recent intervention contact.
