# Supplementary material for: Thermal Imaging for Burn Wound Depth Assessment: A Mixed-Methods Implementation Study
Source: J Clin Med. 2024 Apr 2;13(7):2061. doi: 10.3390/jcm13072061 (PMC11012455; doi:10.3390/jcm13072061)
Supplement: Supplementary file 1 [file jcm-13-02061-s001.zip › jcm-2891005-supplementary.pdf]

Journal of
Clinical Medicine

MDPI

Supplementary File

Eligibility Criteria

Patients of all ages who visited the outpatient clinic within day two to five postburn were eligible for burn wound assessment by thermography. The postburn day was limited to day two to five postburn, as this study used cut-off from a validation study with the LDI as golden standard [15]. Patients with pre-existing vascular comorbidities (e.g., Raynaud, peripheral arterial occlusive disease) or an active wound infection were excluded, as these conditions may affect skin surface temperature.

Imaging Procedure

The thermal images were made by the physician team that worked at the outpatient clinic. During the study period, a single thermography device was used. Heat lamps in the room were turned off, and burn wounds were cleaned and dried. The thermal images were captured at the end of the consultation, before the nurses bandaged the wounds. This allowed the wounds to acclimatize to the stable room temperature. Thermal images were analysed with the FLIR Tools software application (FLIR Systems, Inc, Wilsonville, Ore) for the iPad mini. With this application, temperature differences between burn wound range of interests (ROI) and a reference area can be calculated, which is expressed as ΔT. In a single thermographic image, multiple ROIs can be identified. An area of healthy skin at least 3 cm proximal to the burn wound was chosen as reference area. In cases where the burn wound was located at a distal extremity (e.g., hands or feet), healthy skin on the corresponding contralateral body site was chosen as reference area. Recently published trials demonstrated that ΔT cut-off values enable accurate classification of burn wounds in the healing potential categories <14 and ≥14 days, and <21 and ≥21 days [12–15]. For this study, we used two ΔT cut-off values reported by a study conducted in our own burn centre [15], and extracted two additional ΔT cut-off values from its data using ROC curves (Table S1). All four ΔT cut-off values were based on a 95% specificity and associated sensitivity. See Figure S1 for a thermographic image example.

Rating Process

Two researchers rated the constructs on valence and strength by applying specific rating criteria (Table S2). Valence ratings assess whether a construct has a positive or negative effect on implementation (i.e., is a facilitator or barrier respectively), whereas strength ratings measure the degree of this effect. Constructs were rated on a five-point bipolar scale from −2 to +2, as proposed on the CFIR website [20]. Both researchers individually rated the interviews in a case-oriented approach, after which a variable-oriented approach was used to compare each construct across cases. Disagreements were discussed until consensus was reached.

Table S1. ΔT cut-off values and associated healing potential (HP) categories.

HP-Category	ΔT Cut-Off Value (°C)	Sensitivity (%)	Specificity (%)
≥21 days	<−2.3	30	95
≥14 days	<−0.6	71	95
<21 days	>0.4	51	95
<14 days	>0.6	65	95

Extracted from the study conducted by Carrière et al., 2020. All ΔT cut-off values are based on a specificity of 95%.

Table S2. Criteria used to assign ratings to CFIR constructs.

<table>
<tr><th>Rating</th><th>Criteria</th></tr>
<tr><td>−2</td><td>The construct has a negative influence on the implementation process of the thermal imager. The majority of interviewees describe explicit examples of how the key or all aspects of a construct manifests itself in a negative way.</td></tr>
<tr><td>−1</td><td>The construct is a negative influence on the implementation process of the thermal imager. Interviewees make general statements about the construct manifesting in a negative way but without concrete examples:
The construct is mentioned only in passing or at a high level without examples or evidence of actual, concrete descriptions of how that construct manifests;
There is a mixed effect of different aspects of the construct but with a general overall negative effect;
There is sufficient information to make an indirect inference about the generally negative influence.</td></tr>
<tr><td>0</td><td>A construct has neutral influence if:
It appears to have neutral effect (purely descriptive) or is only mentioned generically without valence;
There is no evidence of positive or negative influence;
Credible or reliable interviewees contradict each other;
There are positive and negative influences at different levels in the organization that balance each other out; and/or different aspects of the construct have positive influence while others have negative influence and overall, the effect is neutral.</td></tr>
<tr><td>+1</td><td>The construct is a positive influence on the implementation process of the thermal imager. Interviewees make general statements about the construct manifesting in a positive way but without concrete examples:
The construct is mentioned only in passing or at a high level without examples or evidence of actual, concrete descriptions of how that construct manifests;
There is a mixed effect of different aspects of the construct but with a general overall positive effect; and/or
There is sufficient information to make an indirect inference about the generally positive influence.</td></tr>
<tr><td>+2</td><td>The construct is a positive influence in the organization, a facilitating influence in work processes, and/or a facilitating influence in implementation efforts. The majority of interviewees describe explicit examples of how the key or all aspects of a construct manifests itself in a positive way.</td></tr>
<tr><td>X</td><td>The interviewee made comments that were purely descriptive. There were no statements that described influence of the construct on implementation.</td></tr>
<tr><td>M</td><td>The interviewee was not asked about the presence and influence of the construct.</td></tr>
<tr><td>*</td><td>Comments were mixed with an overall positive or negative influence on implementation.</td></tr>
</table>

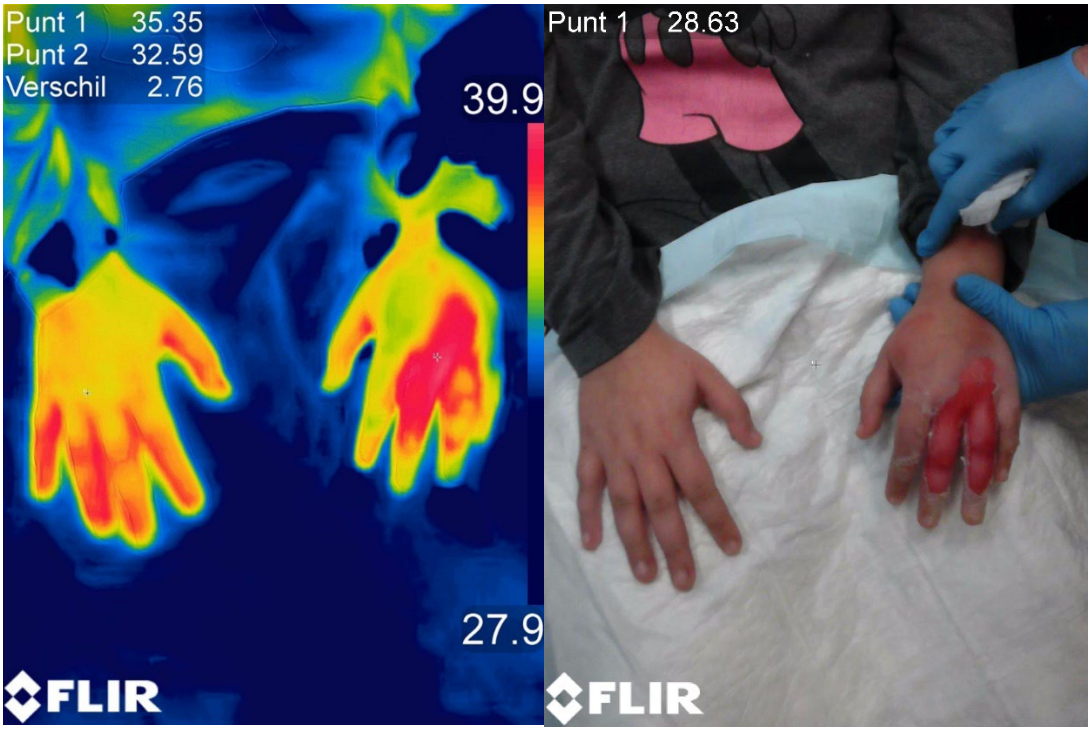

Figure S1. This thermographic image had a ΔT of more than 0.6 °C, corresponding with an expected healing potential of <14 days.
